# Longitudinal monitoring of pulpal and periapical health of adjacent natural teeth during the implant healing period: a prospective cohort study

**DOI:** 10.1186/s12903-026-09211-8

**Published:** 2026-07-14

**Authors:** Helin Kussever, Sehnaz Yilmaz, Bedii Ender Topcu, Cihan Küden

**Affiliations:** 1 Department of Endodontics, Faculty of Dentistry, Cukurova University, Adana, 01380 Turkey; 2 Department of Periodontology, Faculty of Dentistry, Cukurova University, Adana, 01380 Turkey

**Keywords:** Adjacent teeth, Dental implants, Postoperative monitoring, Pulpal health

## Abstract

**Background:**

The biological behavior of natural teeth adjacent to implant sites during surgery and healing remains incompletely understood. This longitudinal cohort study aimed to evaluate changes in pulpal sensibility and periapical status of natural teeth adjacent to an edentulous site planned for implant placement before surgery, during healing, and up to prosthetic rehabilitation.

**Methods:**

Seventy-four teeth adjacent to implant sites in 31 patients were monitored. Clinical evaluations were performed preoperatively and postoperatively at week 1, month 1, and month 3, assessing palpation, percussion, cold testing, electric pulp testing (EPT), and Numerical Pain Rating Scores (NPRS). Radiographic images obtained preoperatively and at 3 months were analyzed for periapical changes. Data were analyzed using Cochran-Mantel-Haenszel, Friedman, and Wilcoxon signed-rank tests. Additionally, linear and logistic mixed-effects models were used to account for intra-patient clustering by treating patient as a random effect (α = 0.05).

**Results:**

Percussion sensitivity was significantly higher at week 1 than preoperatively (*P*=.011), and at month 1 (12.2%) compared to month 3 (0%) (*P*=.046). No significant difference was found in EPT values between preoperative and week 1 (*P*>.05), but week 1 values were significantly higher than month 1 (*P*=.031). Preoperative NPRS scores were significantly lower than week 1, month 1, and month 3 (*P*<.05). One tooth (1.4%) required root canal treatment due to irreversible pulpitis at week 1. Sensitivity analyses using mixed-effects models confirmed the findings, with significant effects for NPRS (β=0.80, *P*<.001) and percussion sensitivity (OR = 5.23, *P*<.001) at the 1-week interval.

**Conclusions:**

Most adjacent teeth maintained pulpal health within 3 months after implant placement. While one case required endodontic treatment, the remaining postoperative changes were transient and consistent with postoperative healing rather than persistent pathological conditions.

**Clinical trial registration:**

This study was registered at ClinicalTrials.gov (NCT06948734) on 29 April 2025.

## Introduction

Dental implant surgery initiates a cascade of biological events within the surrounding osseous tissues, characterized by the activation of inflammatory mediators such as growth factors, cytokines, prostaglandins, and reactive oxygen species [1–4].

These signaling molecules play a pivotal role in orchestrating bone healing and remodeling processes. Although these biochemical responses predominantly occur in the peri-implant region, emerging evidence suggests that their effects may extend beyond the immediate surgical site, potentially influencing the periodontal integrity of adjacent natural teeth [5–7]. Furthermore, it has been suggested that such interactions may indirectly affect the pulpal tissues of adjacent teeth [8–11]. Indeed, several studies involving orthodontic mini-screws have shown that even when there is no direct root contact, close proximity of these external structures may compromise pulpal sensibility [8, 10, 11]. Therefore, monitoring the clinical status of adjacent teeth following implant surgery is crucial to better understand potential biological consequences and to preserve pulpal and periapical health [12–16].

Iatrogenic injury to adjacent teeth during implant placement has been well documented in the literature [9, 12, 13, 17]. However, the potential for pulpal symptoms or biologically mediated alterations in adjacent teeth—even in cases where implants are placed under optimal surgical conditions and at appropriate distances—remains an insufficiently investigated area. Beyond direct mechanical trauma, procedural variables such as overly aggressive osteotomy, the use of non-sharpened drills, or inadequate irrigation may amplify the local inflammatory response within the peri-implant bone, potentially inducing secondary biological stress in adjacent dental structures [18–20]. In certain cases, unusual postoperative symptoms observed at the surgical site during the postoperative healing period may not be fully explained by direct surgical trauma [8, 21]. This raises the possibility of a broader, more complex interplay of biological and mechanical factors affecting adjacent teeth, a subject that continues to generate discussion in the current literature. In this context, the present study aimed to assess the pulpal and periapical status of teeth adjacent to implant placement sites, both before and after surgery, in order to contribute to a deeper understanding of these understudied clinical dynamics. The null hypotheses (H_0_) of this study are as follows: First, there is no significant difference in the clinical parameters of adjacent teeth before and after implant surgery. Second, there is no significant change in the radiological parameters in adjacent teeth between the preoperative and postoperative periods.

## Materials and methods

### Study design, ethical approval, and sample size calculation

This observational study was conducted in the Department of Periodontology and Endodontics at Cukurova University, Faculty of Dentistry, between July 2023 and October 2024. Although non-interventional in nature, the study was registered at ClinicalTrials.gov (NCT06948734) on 29 April 2025 to ensure ethical transparency and traceability, given its prospective patient recruitment and longitudinal follow-up. Designed as a prospective, longitudinal cohort study, it was conducted in accordance with the STROBE guidelines for observational research. The study was conducted in accordance with the principles of the Declaration of Helsinki. Ethical approval was obtained from the Non-Interventional Clinical Research Ethics Committee of Cukurova University (Decision No. 2023/134/53). All procedures were conducted in accordance with the 1964 Declaration of Helsinki and its later amendments. Informed consent was obtained from all participants after providing detailed written and verbal explanations of the study protocol, pain assessment scales, and the potential benefits and risks.

The sample size was calculated using G*Power software (version 3.1, Heinrich Heine University, Germany) based on an effect size of 0.35, α = 0.05, and a power of 0.90. The analysis determined that a minimum of 72 teeth would be required.

### Participant selection

**Fig. 1:**
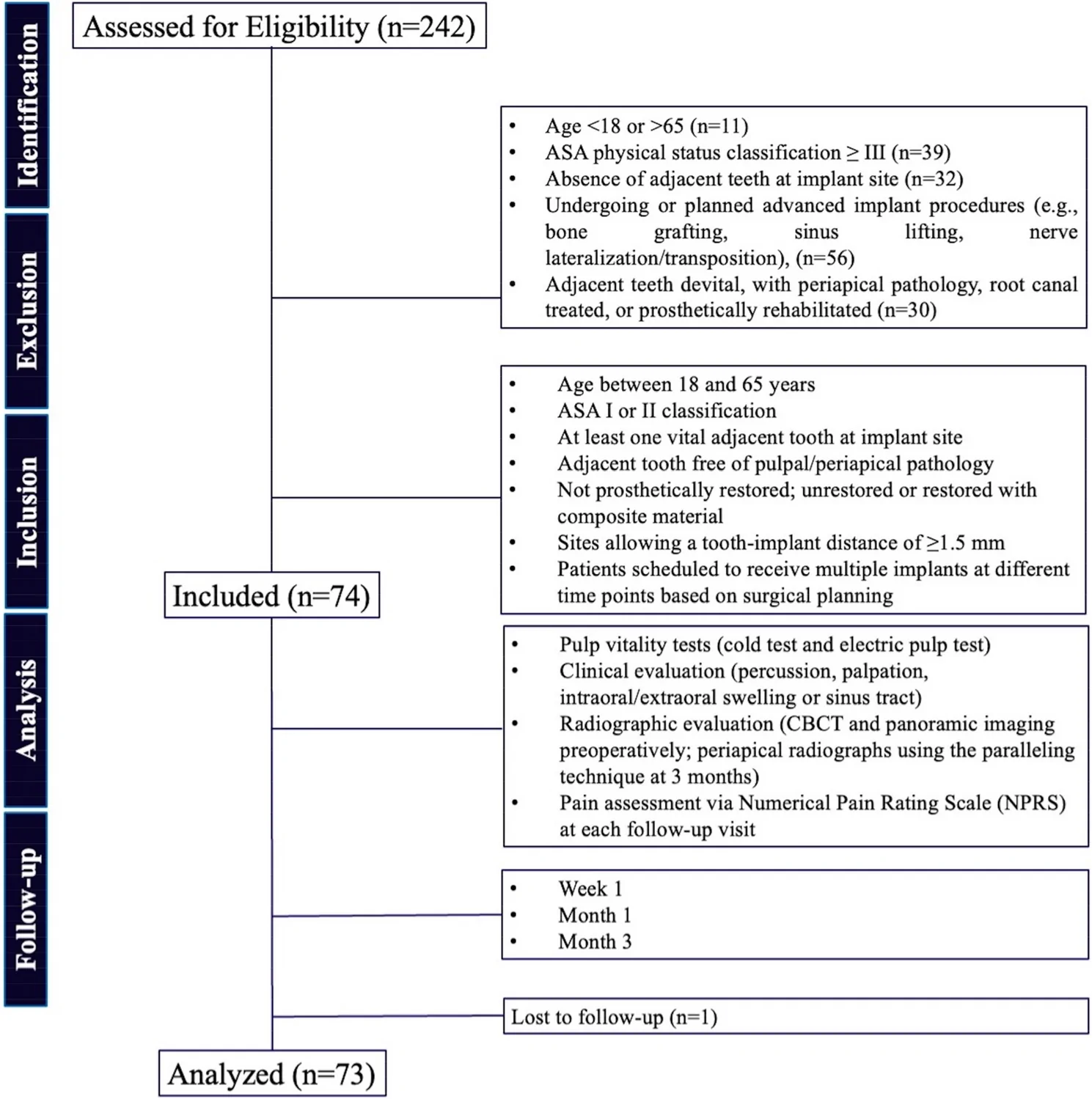
flowchart of the prospective cohort study following STROBE guidelines

A total of 74 teeth adjacent to dental implants from 31 patients were included in this study. Among the 31 patients, 20 (64.5%) contributed multiple teeth (range: 2–6), resulting in 74 analyzed units. These included multiple adjacent teeth from the same site or teeth from separate implants placed at different surgical time points. Figure 1 presents the flowchart of the longitudinal cohort study, outlining the participant selection process and study progression in accordance with STROBE guidelines. Patients were enrolled based on predefined eligibility criteria. Inclusion criteria consisted of individuals aged between 18 and 65 years and classified as ASA I or II (American Society of Anesthesiologists Physical Status Classification System). Eligible participants were scheduled for dental implant placement in single or multiple edentulous areas of the maxilla or mandible. Although multiple implants were placed in some patients, the surgical interventions were performed at different time points based on individualized treatment planning. This approach allowed the adjacent natural teeth to be assessed as having undergone independent surgical exposure after each implant procedure, thereby minimizing the risk of cross-interference. Additionally, included patients had at least one adjacent tooth at the implant site that was vital, showed no signs of pulpal or periapical pathology, had not undergone prosthetic rehabilitation, and was either unrestored or restored with composite restorations.

Exclusion criteria included patients with severe systemic diseases or those lacking adjacent teeth in the region planned for implant placement. Patients who had undergone or were scheduled to undergo advanced implant surgical procedures—such as bone augmentation, grafting, open or closed sinus lifting, or inferior alveolar nerve lateralization or transposition—were also excluded. Furthermore, patients with adjacent teeth that were non-vital, had periapical lesions, had previously undergone root canal treatment, or had been prosthetically rehabilitated in the planned implant site were not included in the study.

### Preoperative evaluation

All patients included in the study underwent clinical and radiographic assessment at the T0 time point (preoperatively), performed by a single clinician (H.K.). Cone-beam computed tomography (CBCT) and panoramic radiographs were obtained for surgical planning and to assess the overall dental condition. Adjacent, permanent, natural teeth in the surgical area were evaluated both clinically and radiographically to ensure pulpal and periapical health. For teeth with existing restorations, the integrity and marginal adaptation of the restorations were also examined and verified to ensure the absence of potential irritants. To confirm pulpal sensibility, cold testing and electric pulp testing (EPT) were performed.

### Implant placement protocol

All implants were placed under local anesthesia using a standardized surgical protocol by a single experienced operator (B.E.T.). In all cases, a full-thickness envelope flap was elevated; a crestal incision was performed at the implant site, and sulcular incisions were extended along the proximal surfaces of the adjacent natural teeth in the implant-adjacent area. Osteotomy sites were prepared using single-use, sterile drilling burs in a sequential manner under copious saline irrigation, in accordance with the manufacturer’s instructions. Implant placement was prosthetically driven, with particular attention to angulation and depth, and performed freehand without the use of a surgical guide.In all cases, a minimum distance of 1.5 mm was maintained between the implant and adjacent natural teeth to preserve periodontal health and alveolar bone. The selected implant sites predominantly consisted of D2–D3 type bone, and the insertion torque ranged between 30 and 40 Ncm. In order to avoid the need for a second surgical procedure and to promote optimal soft tissue healing, healing abutments were preferred over cover screws. Soft tissues were closed using resorbable sutures. Following implant surgery, all patients were prescribed analgesics (ibuprofen) and a chlorhexidine-based mouth rinse (0.12%), and detailed postoperative care instructions were provided. Patients were subsequently scheduled for routine follow-up visits.

### Postoperative evaluation

All clinical postoperative evaluations were conducted at three time points - week 1 (T1), month 1 (T2), and month 3 (T3) - by H.K. Radiographic evaluation, however, was performed only at month 3 (T3), also by H.K. Throughout the study period, no occlusal or restorative interventions were performed on the adjacent teeth, and no signs of occlusal trauma or functional load alterations were observed during baseline or follow-up evaluations, thereby minimizing the potential influence of occlusal factors on the clinical outcomes.

### Clinical examination

To ensure diagnostic consistency and eliminate inter-examiner variability, all clinical assessments and sensibility tests were conducted by a single, experienced endodontist who followed standardized protocols for each diagnostic procedure.

The clinical diagnosis of pulpal and periapical conditions was based on the criteria established by the American Association of Endodontists (AAE). In the event that pulpal pathology was suspected, symptomatic irreversible pulpitis was specifically defined by the presence of spontaneous symptoms and a lingering, painful response to thermal stimulation after the removal of the stimulus.

At each follow-up, teeth adjacent to implants were assessed for tenderness to percussion and palpation. Percussion was performed using a probe handle dropped under its own weight, avoiding additional force. Palpation was conducted using the bidigital technique. Both cold and electric pulp tests were performed during each evaluation to monitor the pulpal condition of teeth adjacent to dental implants. Electric pulp testing was conducted using a digital, monopolar electric pulp tester (Parkell, Inc., Edgewood, NY, USA; range: 0–64) in accordance with the manufacturer’s recommendations. This scale represents a unitless progression of the electrical stimulus, where lower values correspond to a faster pulpal response.

Prior to testing, the teeth were isolated and dried with cotton rolls. A small amount of toothpaste (Parodontax, GlaxoSmithKline, Istanbul, Turkey) was applied to the probe tip, which was then placed on the middle third of the buccal surface of the tooth. To prevent interference with the electric circuit, the operator wore gloves, and the negative electrode (lip clip) was positioned on the patient’s lower lip. The numerical values obtained from EPT were utilized for the longitudinal monitoring of neural response thresholds (quantitative assessment), while the presence or absence of a response was used to confirm pulpal sensibility (qualitative assessment). Corresponding numerical value at the point of perception was recorded.

Cold testing was performed using a refrigerant spray containing a propane-butane mixture (Endo-Frost, Roeko, Langenau, Germany) with a cooling temperature of − 50 °C. The spray was applied to a cotton pellet for three to five seconds, and the pellet was then placed on the middle third of the buccal surface of the test tooth. Care was taken to avoid contact with the gingival tissue during their placement on the buccal surface. In most cases, this elicited a response within five seconds. If no response occurred, the cotton pellet was held in place until the patient reacted.

### Radiographic examination

In addition to preoperative CBCT and panoramic imaging, standardized periapical radiographs were obtained at the 3-month follow-up using the paralleling technique. This technique, which is widely accepted for longitudinal evaluations, allows for accurate and reproducible linear assessments. Radiographic evaluation focused on two primary parameters: the presence or absence of periapical radiolucency in the adjacent teeth, and the continuity of the adjacent tooth’s lamina dura. These parameters were compared between baseline and the 3-month follow-up in order to detect any pathological changes.

Radiographic periapical status was evaluated based on the modified Strindberg criteria, focusing on the continuity of the lamina dura and the absence of periapical radiolucency [22]. Assessments were performed by a single, experienced endodontist to ensure diagnostic consistency. Any tooth showing widening of the periodontal ligament space or a new radiolucent area was recorded as having a detectable periapical change.

### Pain assessment

At each visit, patients were asked to evaluate the intensity of any pain experienced in adjacent teeth using a Numerical Pain Rating Scale (NPRS), an 11-point scale ranging from 0 (no pain) to 10 (worst imaginable pain). For interpretive purposes, scores of 1–3 were considered mild, 4–6 moderate, and 7–10 severe pain.

This self-reported assessment was completed by the patients themselves prior to any clinical procedures, such as palpation or percussion testing, and was conducted preoperatively as well as at each postoperative follow-up (week 1, month 1, and month 3).

### Statistical analysis

Statistical analyses were performed using SPSS version 22 (IBM Corp., Armonk, NY, USA). Based on data measurement levels and normality assessments, the Cochran–Mantel–Haenszel and Friedman tests were applied. Pairwise comparisons between time points were conducted using the Wilcoxon signed-rank test. To account for potential intra-patient clustering, sensitivity analyses were performed using linear mixed-effects models and mixed-effects logistic regression, treating the patient as a random effect to control for the dependency between multiple teeth from the same individual. All analyses were conducted with a 95% confidence interval (α = 0.05).

## Results

The study population consisted of 31 patients with a mean age of 48.5 years (SD: 9.3), the majority of whom were female (64.5%). A total of 74 adjacent teeth were included in the analysis, of which 50% were premolars, 31.1% molars, and 18.9% incisors. Maxillary teeth accounted for 58.1% of the sample, while 41.9% were located in the mandible. These demographic and anatomical characteristics are detailed in (Table 1).

**Table 1:** Baseline demographic characteristics of the study population and distribution of adjacent teeth

Variable	Value
Age (years), mean (SD)	48.5 (9.3)
Gender Pat. count (%)	
Female	20 (64.5%)
Male	11 (35.5%)
Included teeth count (%)	
Incisors	14 (18.9%)
Premolars Molars	37 (50%) 23 (31.1%)
Region of the teeth (%)	
Maxillary Mandibular	43 (58.1%) 31 (41.9%)

SD: Standard deviation; Pat: Patient

Notably, the only tooth exhibiting severe sensitivity at T1 was diagnosed with irreversible pulpitis and was excluded from further analysis following endodontic intervention.

This affected tooth was the maxillary right canine, which had a large restoration but was asymptomatic prior to surgery. Preoperative clinical and radiographic evaluations confirmed its pulpal and periapical health.

### Clinical course and adverse events

Of the 74 monitored teeth, 73 (98.6%) maintained pulpal health and remained in the study throughout the 3-month follow-up. One case (1.4%) involved a maxillary right canine that had a large composite restoration but was completely asymptomatic prior to surgery. Preoperative clinical and radiographic evaluations had confirmed its initial pulpal and periapical health, with a positive cold response and no signs of pathology. However, at the 1-week follow-up (T1), this tooth presented with symptoms leading to a clinical diagnosis of symptomatic irreversible pulpitis. The diagnosis was established based on AAE criteria, specifically the presence of spontaneous paroxysmal pain (NPRS = 8) and a characteristically intense, lingering painful response to cold stimulation (lingering for more than 30 s). Sensitivity to palpation and percussion was also noted at this visit, suggesting associated symptomatic apical periodontitis. Following the initiation of root canal treatment at the end of the T1 visit, the acute symptoms resolved within one week. The tooth remained completely asymptomatic at the treatment completion and during the subsequent 1-month (T2) and 3-month (T3) follow-up visits. This tooth was excluded from subsequent longitudinal statistical analyses to maintain the integrity of the data related to healthy adjacent teeth (*N* = 73 for T2 and T3).

### Palpation sensitivity in adjacent teeth over time

Palpation sensitivity results across all follow-up intervals are summarized in (Table 2). At baseline (T0), all teeth (100%) were asymptomatic. At T1, a positive response was recorded in a single tooth (1.4%)—the case of irreversible pulpitis described in the ‘Clinical Course’ section—which was subsequently excluded from further longitudinal analysis. Among the remaining 73 teeth, three (4.1%) exhibited transient palpation sensitivity at T2, all of which resolved completely by T3. No statistically significant differences were found between consecutive time points (T1–T2: *P* = .083; T2–T3: *P* = .083).

**Table 2:** Longitudinal distribution and pairwise comparisons of palpation sensitivity in adjacent teeth across four evaluation time points

Time Point	Positive (*n*, %)	Negative (*n*, %)	*P* value
Preoperative (T0)	0 (0.0%)	74 (100.0%)	
Week 1 (T1)	1 (1.4%)	73 (98.6%)	
T0 vs. T1			0.317
Month 1 (T2)	3 (4.1%)	70 (95.9%)	
T1 vs. T2			0.083
Month 3 (T3)	0 (0.0%)	73 (100.0%)	
T2 vs. T3			0.083

Statistical significance was set at 0.05

### Percussion sensitivity in adjacent teeth over time

Changes in percussion sensitivity over time are summarized in (Table 3). A statistically significant increase in positive responses was observed from baseline (1.4%) to T1 (12.2%, *P* = .011). This T1 assessment included the single case of symptomatic irreversible pulpitis, which exhibited severe percussion sensitivity before being excluded from the subsequent follow-up intervals. For the remaining 73 teeth, the proportion of positive responses showed a gradual decline at T2 (5.5%), and the subsequent decrease to 0% at T3 reached statistical significance (*P* = .046). 

**Table 3:** Changes in percussion sensitivity of teeth adjacent to implants: Frequency distribution and statistical significance over 3 months

Time Point	Positive (*n*, %)	Negative (*n*, %)	*P* value
Preoperative (T0)	1 (1.4%)	73 (98.6%)	
Week 1 (T1)	9 (12.2%)	65 (87.8%)	
T0 vs. T1			0.011
Month 1 (T2)	4 (5.5%)	69 (94.5%)	
T1 vs. T2			0.206
Month 3 (T3)	0 (0.0%)	73 (100.0%)	
T2 vs. T3			0.046

Statistical significance was set at 0.05

### Evaluation of electric pulp test (EPT) responses over time

The distribution of numerical responses to electric pulp testing over time, including mean, minimum, maximum, and median values, was summarized in (Table 4). Although the mean score decreased from 6.7 at T0 to 6.5 at T1, this change was not statistically significant (*P*= .264). A significant reduction was observed between T1 and T2, with the mean score dropping to 6.0 (*P* = .031). No significant difference was found between T2 and T3, despite a further decline to 6.0 (*P* = .982). Despite this statistical significance, the absolute numerical change was small (e.g., from 6.7 to 6.0). Given that EPT reflects pulpal neural sensibility rather than true vascular vitality, these findings were reported as shifts in neural response thresholds and were interpreted strictly in conjunction with other clinical and radiographic parameters to avoid overstating their biological significance.

**Table 4:** Electric pulp test (EPT) response thresholds over time in adjacent teeth and pairwise comparisons

Time Point	*n*	Mean	Median	Minumum	Maximum	SD	*P* value
Preoperative (T0)	74	6.7	6.0	2.0	15.0	2.8	
Week 1 (T1)	74	6.5	6.0	3.0	11.0	1.8	
T0 vs. T1							0.264
Month 1 (T2)	73	6.0	6.0	3.0	10.0	2.0	
T1 vs. T2							0.031
Month 3 (T3)	73	6.0	6.0	2.0	10.0	2.0	
T2 vs. T3							0.982

SD: Standard deviation. Statistical significance was set at 0.05

### Evaluation of cold test responses over time

At all evaluation time points, 100% of the adjacent teeth (74/74) remained cold-test positive, confirming that pulpal sensibility was preserved throughout the healing period. As detailed in the “Clinical Course and Adverse Events” section, 73 teeth (98.6%) consistently exhibited normal, transient positive responses. The single case diagnosed with symptomatic irreversible pulpitis at T1 was characterized by an abnormal, lingering response to cold, while maintaining its overall sensibility. Given the uniform preservation of sensibility across the cohort and the lack of longitudinal variation in responses among the remaining 73 teeth, these findings are reported qualitatively.

### Evaluation of pain levels using the numeric pain rating scale (NPRS)

Postoperative pain levels in adjacent teeth across the follow-up period are summarized in (Table 5). At T1, a severe pain score (NPRS = 8) was recorded in the previously described case of irreversible pulpitis. For the remaining teeth, NPRS scores typically ranged between 0 and 4. All patients had a preoperative NPRS score of 0, which was significantly lower than the scores recorded at T1 (*P* = .001). Furthermore, the mean NPRS score at T1 was significantly higher than at T2 (*P* = .001), and scores at T2 remained significantly higher than those at T3 (*P* = .002). By T3, pain had resolved in 90% of the adjacent teeth, with only mild symptoms persisting in the remainder.

While the mean NPRS score at the 1-week interval was low (0.80 ± 1.28), it represented a significant clinical shift, with 46% of the adjacent teeth (34/74) exhibiting measurable discomfort (NPRS > 0). The linear mixed-effects model identified a statistically robust effect size (β = +0.80, *P* < .001), reflecting a clear departure from the baseline where 100% of the teeth were asymptomatic. This indicates that although the pain was mostly mild, it was a consistent biological response observed in a substantial proportion of the cohort following surgical stress.

**Table 5:** Distribution of numeric pain rating scale (NPRS) scores in adjacent teeth across follow-up time points and pairwise comparisons

Time Point	*n*	Mean	Median	Minimum	Maximum	SD	*P* value
Preoperative (T0)	74	0.0	0.0	0.0	0.0	0.0	
Week 1 (T1)	74	0.8	0.0	0.0	8.0	1.28	
T0 vs. T1							0.001
Month 1 (T2)	73	0.34	0.0	0.0	4.0	0.71	
T1 vs. T2							0.001
Month 3 (T3)	73	0.1	0.0	0.0	1.0	0.3	
T2 vs. T3							0.002

SD: Standard deviation. Statistical significance was set at 0.05

Categorical analysis of pain intensity based on NPRS scores is presented in (Table 6). Across all postoperative time points, the majority of adjacent teeth reported no pain, with the highest proportion observed at month 3 (90%). Mild pain (NPRS 1–3) was most frequently reported at week 1 (45%) and showed a gradual decline at month 1 (23%) and month 3 (10%). Moderate pain and severe pain were uncommon throughout the follow-up period.

**Table 6:** Frequency and percentage distribution of pain intensity categories across follow-up intervals based on NPRS scores

Time point	Mild (1–3)	Moderate (4–6)	Severe (7–10)	No pain (0)	Total (*n*)
T0	0 (0%)	0 (0%)	0 (0%)	74 (100%)	74
T1	33 (45%)	0 (0%)	1 (1%)	40 (54%)	74
T2	17 (23%)	1 (1%)	0 (0%)	55 (76%)	73
T3	7 (10%)	0 (0%)	0 (0%)	65 (90%)	73

T0: Preoperative, T1: Week 1, T2: Month 1, T3: Month 3

### Sensitivity Analysis for Intra-patient Clustering

To account for the potential dependency of multiple teeth within the same individual (intra-patient clustering), additional advanced statistical models—specifically Linear Mixed-Effects Models (LMM) for continuous data and Mixed-Effects Logistic Regression for binary data—were performed. In these models, the patient was included as a random effect to control for intra-individual variability, while the repeated measurements across time points were modeled as fixed effects.

As summarized in Table 7, the results of this sensitivity analysis were entirely consistent with the primary tooth-level analyses regarding the direction and significance of all clinical findings. While the mean NPRS score at the 1-week interval was low (0.80 ± 1.2), the linear mixed model identified a significant effect size (β = +0.80, *P* < .001), representing a clear departure from the baseline where all monitored teeth were completely asymptomatic. This shift indicates that while the discomfort was clinically mild, it represented a consistent and statistically robust biological response across the affected cohort.

Percussion sensitivity showed the highest clinical shift at the 1-week interval (OR = 5.23, *P* < .001), which decreased notably by the third month. The EPT threshold showed a minimal but statistically significant numerical reduction by the 3-month follow-up (β = -0.66, *P* = .003), while palpation sensitivity remained statistically unchanged throughout the study (*P* = .341). These advanced models confirm that the study’s conclusions are robust and were not influenced by the dependency between teeth within the same patient.

The reliability of the EPT findings was further supported by high intra-examiner consistency and the stability of the median values (Median = 6), which remained constant across all time points. While the linear mixed model identified a minimal numerical reduction in the EPT threshold by T3 (β = −0.66, *P* = .003), the results showed a 100% correlation with cold test findings regarding pulpal sensibility. This confirms that the minor numerical fluctuations in EPT thresholds did not signify a functional decline in pulpal health. Palpation sensitivity remained statistically unchanged throughout the study (*P* = .341).

**Table 7:** Sensitivity analysis of postoperative clinical outcomes in adjacent teeth using linear and logistic mixed-effects modeling to account for intra-patient clustering

Outcome measure	Statistical model	Effect size	95% CI	*P* value	Comparison with primary analysis
NPRS	Linear Mixed Model	β = +0.80 (at T1)	0.45–1.15	< 0.001	Consistent
EPT Threshold	Linear Mixed Model	β = -0.66 (at T3)	-1.09 – -0.22	0.003	Consistent
Percussion	Mixed-effects Logistic	OR = 5.23 (at T1)	2.42–11.30	< 0.001	Consistent
Palpation	Mixed-effects Logistic	OR = 1.32 (Overall)	0.74–2.36	0.341	Consistent

*Abbreviations*: *NPRS *Numeric Pain Rating Scale, *EPT *Electric Pulp Test, *CI *Confidence Interval, *β *Regression Coefficient, *OR *Odds Ratio. Statistical significance was set at 0.05

## Discussion

Dental implant surgery, while widely accepted and routinely performed, raises important questions regarding its potential impact on the pulpal sensibility and clinical status of adjacent teeth. This study contributes to this underexplored area by systematically evaluating both clinical and radiological parameters in adjacent teeth before and after implant surgery. In line with the study’s null hypotheses, clinical parameters—including EPT scores and percussion sensitivity findings—showed statistically significant differences following implant placement, leading to the rejection of the first null hypothesis. In contrast, no significant changes were observed in the radiological parameters, such as periapical status and the presence of pathology, and thus the second null hypothesis was not rejected.

Maintaining the health of adjacent teeth is essential not only for long-term treatment success but also for preserving patient comfort and function. Despite advances in surgical techniques and planning technologies, limited data exist regarding the biological and clinical changes that may occur in teeth adjacent to implant placement. In this context, the present study investigated the pulpal and periapical status of teeth adjacent to the implant site by performing cold and electric pulp tests, percussion and palpation assessments, and radiographic evaluations at multiple postoperative time points. The findings suggest that although clinical signs of altered pulpal response or sensitivity may occur postoperatively, these changes do not necessarily correspond to radiographically detectable pathology—except for one case, which should be viewed as an isolated adverse event rather than a generalized biological risk.

Following dental implant surgery, the healing processes of the surrounding hard and soft tissues progress in a synchronized and dynamic manner [23]. Within the first postoperative week, histological signs of early bone formation emerge as the initial blood clot and granulation tissue are gradually replaced by a provisional connective tissue matrix rich in fibroblast-like mesenchymal cells [4, 24]. Subsequently, woven bone becomes apparent in the surgical area and progressively matures, occupying an increasing proportion of the site. According to the literature, by the end of the fourth week, woven bone comprises approximately 30% of the surgical field [3]. Over the following 1 to 3 months, this newly formed bone undergoes gradual remodeling and is replaced by lamellar bone [2, 7]. Beginning at the end of the third month, the bone remodeling phase represents a lifelong physiological process. These stages constitute the biological phases of osseointegration [3]. Accordingly, in the present study, follow-up sessions were scheduled at postoperative week 1, month 1, and month 3 to evaluate potential clinical changes in adjacent teeth, while radiographic assessment was performed only at the 3-month follow-up. These time points were selected based on their correspondence to the key biological phases of osseointegration and their relevance to the possible onset of pulpal or periapical changes.

Moving beyond this specific adverse event, the broader clinical data across the entire study sample requires a careful interpretation of its practical implications. In this context, it is important to distinguish the observed statistical significance from clinical relevance. Although parameters such as EPT thresholds, percussion sensitivity, and NPRS scores showed significant longitudinal shifts, the absolute numerical changes remained notably low. These results suggest that while the adjacent dentition undergoes a detectable biological response to the surgical stress of implant placement, this response is typically self-limiting and may be indicative of a transient pulpal reaction that does not reach a threshold of clinical pathology in the majority of instances.

The placement of orthodontic mini-screws offers a relevant biological parallel to dental implants regarding their impact on adjacent dentition [11, 15]. Studies indicate that even without direct root contact, the proximity of these devices can alter local microcirculation and compromise pulpal sensibility [10, 14, 16]. While such changes are frequently transient, some instances progress to irreversible damage requiring endodontic intervention [11]. For instance, pulpal degeneration necessitating therapy has been reported where mini-implants were placed near anterior teeth, despite a lack of radiographic root injury [8]. These findings reinforce our observation that the biological proximity of an external device, rather than direct mechanical trauma alone, can trigger responses ranging from transient inflammation to irreversible pulpitis.

The majority of studies investigating the biological effects of dental implants on adjacent teeth are retrospective or cross-sectional in design, with follow-up periods typically ranging from one to ten years [20, 25]. These studies generally focus on broad clinical outcomes such as caries incidence or the need for endodontic treatment. In contrast, the present study systematically assessed the pulpal status of all adjacent teeth using objective, physiology-based diagnostic methods—including electric pulp testing, cold testing, percussion, and palpation—at defined postoperative intervals. All included teeth were clinically and radiographically healthy at baseline, allowing any postoperative changes to be interpreted as deviations from a stable initial condition. This approach, applied to a heterogeneous sample reflecting real-world variability in tooth location and restorative history, enhances the clinical relevance of the findings. To date, no comparable study has been identified in the literature that monitors such a comprehensive set of clinical parameters throughout the healing period.

The relationship between implant-to-tooth distance and pulpal health remains a critical focus in recent literature. While our study utilized a minimum clearance of 1.5 mm, other researchers have explored the consequences of narrower proximities. For instance, Hamdoon et al. [13] reported transient percussion sensitivity in 33% of cases when implants were placed within 0.5 mm of adjacent roots. Supporting this, Han et al. [21] emphasized through a retrospective analysis that while proximity is a significant variable, many adjacent teeth can maintain vitality even with reduced clearance, provided that direct trauma is avoided. Furthermore, Ribas et al. [17] highlighted that positioning errors are common and can impact adjacent anatomical structures, underlining the importance of precise 3D assessment during planning. In contrast to these high-risk scenarios, our study observed a significantly lower incidence (12.2%) of transient percussion sensitivity at the one-week interval with a ≥ 1.5 mm safety distance. This discrepancy supports the hypothesis that increasing the implant-to-tooth spacing may directly reduce early inflammatory biological responses. This is particularly relevant given that recent systematic reviews have reported that teeth adjacent to implants have a 2.12 times higher risk of developing periapical radiolucency compared to implant-free regions, with a reported prevalence of 2.55% [18]. However, as the overall quality of evidence in such studies is often rated as “low,” these findings should be interpreted with caution. Our results also align with the more conservative clinical outcomes reported by Yi et al. [9], who found that only 8.3% of teeth required endodontic treatment even at distances less than 1 mm. Collectively, these comparisons reinforce the clinical importance of maintaining adequate spacing and utilizing precise imaging to preserve the long-term health of the adjacent dentition.

Regarding the biological mechanisms, several studies support the mechanistic speculation that thermal energy generated during implant surgery may influence surrounding vital tissues [19, 26–28]. While the exposure of bone to 47 °C or a pulpal temperature rise of 5.5 °C represents established physiological thresholds for damage [29], the observed data in our specific cohort—characterized by a low-magnitude, transient increase in percussion sensitivity (12.2% at T1)—suggests a sub-clinical inflammatory response rather than definitive thermal injury. Therefore, while thermal stress and mechanical vibration remain plausible hypotheses for the observed changes, they should be viewed as theoretical frameworks that warrant further investigation with more direct assessment tools.

Within this framework, the single case of symptomatic irreversible pulpitis (1.4%) identified at the one-week follow-up warrants closer examination. At baseline, the involved tooth (maxillary right canine) exhibited a healthy, positive response to cold testing and a stable EPT threshold (Median = 6), identical to the surrounding dentition. Postoperative radiographic verification confirmed an actual implant-to-tooth distance of 1.7 mm, successfully maintaining the ≥ 1.5 mm safety clearance. While causality cannot be definitively established, the temporal association between the surgery and symptom onset (NPRS = 8, lingering cold response) raises a clinical concern. The presence of a stable composite restoration (placed > 6 months prior) may have acted as a potential modifying factor, though subclinical pulpal changes not detectable during baseline assessment cannot be entirely excluded. This instance highlights the importance of monitoring adjacent dentition during the healing phase but does not establish a generalized biological risk of implant-induced pulpal damage.

Dental implants lack a periodontal ligament and, unlike natural teeth, have limited physiologic mobility—reportedly around 5 μm under occlusal loading, compared to approximately 28 μm in natural teeth and they also do not undergo mesial drift [30, 31]. This ankylosed-like behavior contributes to interproximal contact loss between implants and adjacent teeth, particularly on the mesial side of mandibular restorations [31–33]. Such contact loss is commonly observed within the first three months after crown placement and tends to increase over time [34]. Clinically, it may lead to food impaction, discomfort, soft tissue inflammation, and even caries or alveolar bone loss in adjacent teeth [5, 20, 31, 35]. Given that the etiology of this condition largely stems from the biomechanical differences between implants and natural teeth, implant-supported restorations were excluded from direct evaluation in this study. Instead, the focus was placed exclusively on the biological and endodontic status of adjacent natural teeth, to avoid confounding and maintain interpretive clarity.

This study has several limitations that warrant consideration. Primarily, its single-center design and the lack of blinding during clinical assessments represent methodological constraints. While a multicenter investigation might enhance generalizability, the current approach ensured high internal validity through strictly standardized protocols and objective digital diagnostic tools, effectively mitigating potential observer bias. A significant methodological strength is that each tooth served as its own internal control, with preoperative baseline (T0) data providing a stable comparator for assessing longitudinal shifts. This prospective approach minimizes the confounding variables often encountered in inter-individual or contralateral comparisons. From a statistical and temporal perspective, although analysis was primarily conducted at the tooth level, post-hoc sensitivity analyses using mixed-effects modeling confirmed that intra-patient clustering did not influence the robustness of our conclusions. Regarding the evaluation period, the 3-month follow-up was selected to correspond with the key phases of osseointegration; however, this duration may be insufficient to detect long-term periapical pathologies that manifest only after functional prosthetic loading. Clinical variables such as restorative history and tooth type also require careful interpretation. While 36% of the teeth had pre-existing restorations, their uniform maintenance of sensibility suggests that restorative status was not a decisive factor for adverse pulpal outcomes under the surgical conditions of this study. Furthermore, the high overall survival rate of adjacent teeth (98.6%) precluded a detailed subgroup analysis of variables like jaw location or tooth type. Finally, while we utilized standardized sensibility tests, the small numerical fluctuations in EPT values should be interpreted with caution, as they may not represent direct changes in vascular vitality. Future multicenter trials incorporating more direct physiological tools, such as laser Doppler flowmetry or pulse oximetry, are necessary to confirm these preliminary longitudinal findings.

## Conclusions

In conclusion, within the pre-loading healing period evaluated in this study, the majority of adjacent teeth preserved pulpal health within 3 months after implant placement performed with an implant-to-tooth distance of approximately 1.5 mm. While one case required endodontic treatment, postoperative increases in percussion sensitivity and pain were generally transient and resolved over time, indicating that such postoperative clinical alterations in adjacent teeth are usually self-limiting.

## Data Availability

The datasets analyzed during the current study are available from the corresponding author on reasonable request due to privacy reasons.

## Abbreviations

EPT: Electric pulp testing
NPRS: Numerical pain rating scores
CBCT: Cone-beam computed tomography
T0: Preoperative
T1: Week 1
T2: Month 1
T3: Month 3
